# Educational attainment of individuals with lymphangioleiomyomatosis is a determinant of timely diagnosis and treatment uptake

**DOI:** 10.1186/s13023-026-04284-8

**Published:** 2026-02-23

**Authors:** Marina K. Holz

**Affiliations:** Graduate School of Biomedical Sciences, New York Medical College, Valhalla, NY 10595 USA

**Keywords:** Lymphangioleiomyomatosis (LAM), Rare diseases, Tuberous Sclerosis Complex (TSC), Patient outcomes, Education

## Abstract

**Background:**

Lymphangioleiomyomatosis (LAM) is a rare, progressive lung disease predominantly affecting women. Despite the availability of FDA-approved treatments, mTOR inhibitors like sirolimus or everolimus, significant disparities persist in early diagnosis, treatment utilization, and patient outcomes. The goal of this study is to examine how key socioeconomic factors affect diagnostic delay and treatment uptake in LAM. It was hypothesized that higher educational attainment is associated with earlier diagnosis and greater likelihood of treatment utilization due to its association with health literacy.

**Results:**

To investigate the impact of socioeconomic factors, we analyzed data from the LAM Patient Research Priorities (LAM-PREP) study, a cross-sectional survey of LAM patients representing ~ 20% of known cases globally. 624 participants completed the survey and 566 provided complete education data and were therefore included in analyses. Multiple regression and logistic regression analyses were performed to examine the associations between educational attainment and diagnostic timing, as well as treatment utilization, controlling for age and disease severity. Among 566 patients with complete educational data, there was a 10-year average delay between self-reported symptom onset (mean age 25.0 ± 12.7 years) and diagnosis (mean age 35.1 ± 13.5 years). Educational attainment emerged as a significant predictor of diagnostic delay. Patients with bachelor’s degrees were diagnosed 4.7 years earlier than those without a bachelor’s degree (95% CI: -7.4 to -2.0, *p* = 0.001). Only 53% of patients reported using mTOR inhibitors. Educational attainment was a significant predictor of treatment utilization, with patients holding a bachelor’s degree having 1.75 times higher adjusted odds of using mTOR inhibitors (AOR 1.75, 95% CI: 1.13–2.73, *p* = 0.013), and those with graduate degrees having 2.21 times higher odds (AOR 2.21, 95% CI: 1.41–3.45, *p* = 0.001).

**Conclusions:**

Educational attainment was significantly associated with both the timing of diagnosis and the likelihood of treatment uptake in LAM patients. These findings highlight the need for targeted interventions to address healthcare disparities in LAM care.

## Background

Understanding social determinants of health (SDOH) in rare diseases is critical for developing effective behavioral and treatment interventions that improve patient outcomes. Lymphangioleiomyomatosis (LAM) is a rare, progressive lung disease that almost exclusively affects women, characterized by cystic lung destruction and declining pulmonary function [1, 2]. LAM can occur sporadically (S-LAM) or in the context of the genetic disease Tuberous Sclerosis Complex (TSC). LAM is caused by mutations in the *TSC1/2* genes, leading to abnormal activation of the mechanistic target of rapamycin (mTOR) pathway [1]. While FDA-approved mTOR inhibitors, such as sirolimus or everolimus, stabilize or reduce lung function decline [3], significant disparities persist in early diagnosis, treatment utilization, and long-term outcomes. In addition to being a rare disease, LAM has nonspecific early symptoms (such as dyspnea or pneumothorax), and the need for specialized pulmonary or radiologic evaluation [3]. This means that the patients’ ability to recognize symptoms, seek appropriate evaluation, and access disease-modifying therapies is shaped by socioeconomic constraints that influence healthcare navigation, provider engagement, and access to specialty care.

No studies have systematically explored how socioeconomic factors affect disease management and outcomes in individuals with LAM. A large scoping review of 136 studies of other rare diseases demonstrated that patients frequently experience inequities in diagnosis and care, including delayed diagnosis, insufficient clinician knowledge, limited service availability, and disparities associated with socioeconomic status, race/ethnicity, and geographic location [4]. Because rare diseases require more complex navigation, any underlying socioeconomic limitations have a magnified impact, affecting access to specialized centers and coordination of care [5]. While most studies show that the clinicians’ knowledge gaps delay the diagnosis and appropriate treatment of rare diseases [4], individuals with lower education may not recognize early symptoms or may struggle to obtain information about the diagnostic process and treatment plans [4, 6]. The goal of this study is to examine how key socioeconomic factors affect diagnostic delay and treatment uptake in LAM. It was hypothesized that higher educational attainment is associated with earlier diagnosis and greater likelihood of treatment utilization due to its relationship with health literacy and levels of health [7].

The recently completed LAM Patient Research Priorities (LAM-PREP) study addresses this gap with a robust dataset of 624 LAM patients, representing approximately 20% of known LAM patients globally [8]. This dataset includes detailed sociodemographic information, including educational attainment of patients with LAM, as well as clinical characteristics, and patient-reported outcomes, which enabled me to conduct analyses rarely feasible in the context of such a rare condition. By analyzing data from the LAM-PREP study, this research aims to identify disparities and inform strategies to improve LAM care.

## Methods

### Dataset

LAM-PREP is a cross-sectional survey conducted in 2024 and described in [8]. The survey, with the primary objective of ranking LAM patient research priorities, was created in SurveyMonkey (SurveyMonkey.com) and distributed via email and social media channels in collaboration between New York Medical College and the LAM Foundation. The survey was open from 03/06/2024 to 05/31/2024 and received 624 responses from LAM patients, of which 50.7% were from the United States and 49.3% were international, and collected data on demographics, clinical history, and treatment use. 566 participants provided complete education data and were therefore included in analyses. The full description of the respondent demographic and clinical characteristics is described in [8].

### Variables

The primary exposure variable was educational attainment, categorized as less than bachelor’s degree (high school, technical/trade/vocation training, some college or associate degree), bachelor’s degree, and graduate degree (master’s or doctorate), and are described in Table 1. “Less than a bachelor’s degree” served as the reference category in all regression analyses. Outcomes included self-reported age at diagnosis, age at symptom onset, and current use of mTOR inhibitors (such as sirolimus or everolimus). Covariates included age group, disease severity, type of LAM (S-LAM or TSC-LAM), income, race/ethnicity, and health insurance status. Logistic regression was used to examine associations with mTOR inhibitor utilization.

### Statistical analysis

Descriptive statistics were used to summarize participant characteristics. Multiple linear regression was used to examine associations between education and continuous outcomes, while logistic regression was used for binary outcomes. All analyses were conducted using Stata SE version 17.

## Results

### Model testing and variable selection

I initially fit a logistic regression model with education as the sole predictor to assess its unadjusted association with mTOR inhibitor use. To improve explanatory power and control for potential confounding, I sequentially added relevant covariates: age group, disease severity, type of LAM, income, race, and insurance status. Clinically, age correlates with disease stage at presentation and cumulative symptom burden [9]. Disease severity represents one of the strongest clinical predictors of mTOR inhibitor use, which are recommended for patients with moderate or severe impairment in lung function or evidence of disease progression, making disease severity a direct clinical indication for treatment [3]. Because severity may correlate with healthcare engagement, frequency of specialist visits, and likelihood of being offered treatment, adjusting for severity ensures that the association between education and treatment utilization is not driven by differences in clinical need. LAM type was included because S-LAM and TSC-LAM differ in typical age of onset; individuals with TSC-LAM are often diagnosed earlier due to ongoing surveillance for TSC manifestations, whereas those with sporadic disease may present only after respiratory symptoms develop [2]. Income, race/ethnicity, and insurance status were evaluated because of their known associations with healthcare access and health‑system navigation [5]. “Less than a bachelor’s degree” served as the reference category in all regression analyses. Model comparisons were based on likelihood ratio (LR) tests, Akaike Information Criterion (AIC), and Bayesian Information Criterion (BIC), calculated on the same estimation sample. The education-only model had poor fit (Pseudo R² = 0.015), while adding age group and disease severity substantially improved fit (Pseudo R² = 0.115; AIC decreased by ~ 64 points). Including income, race, and health insurance status yielded only a marginal improvement in log-likelihood and increased model complexity, resulting in worse AIC/BIC. Education coefficients changed by less than 10% across specifications, indicating minimal confounding by these additional variables. Adding LAM type improved fit, however sporadic vs. TSC-associated LAM did not differ after controlling for age and severity, while patients whose LAM type was unknown were much less likely to be on mTOR inhibitors. Based on these criteria, I selected the model to include education, age group, and disease severity as the final specification for parsimony and interpretability, with statistical significance set at *p* < 0.05. The final model demonstrated good overall fit (LR χ²  = 89.94, *p* < 0.001) and explained substantially more variation than the education-only model (Pseudo R² = 0.115 vs. 0.015). Compared to a fully adjusted model with income, race, and insurance, the selected model achieved a lower AIC and BIC, indicating superior balance of fit and complexity. Education effects remained stable across specifications, and disease severity showed a strong graded association with mTOR inhibitor use, supporting the clinical plausibility of the final model.

### Diagnostic delay across age groups

**Fig. 1:**
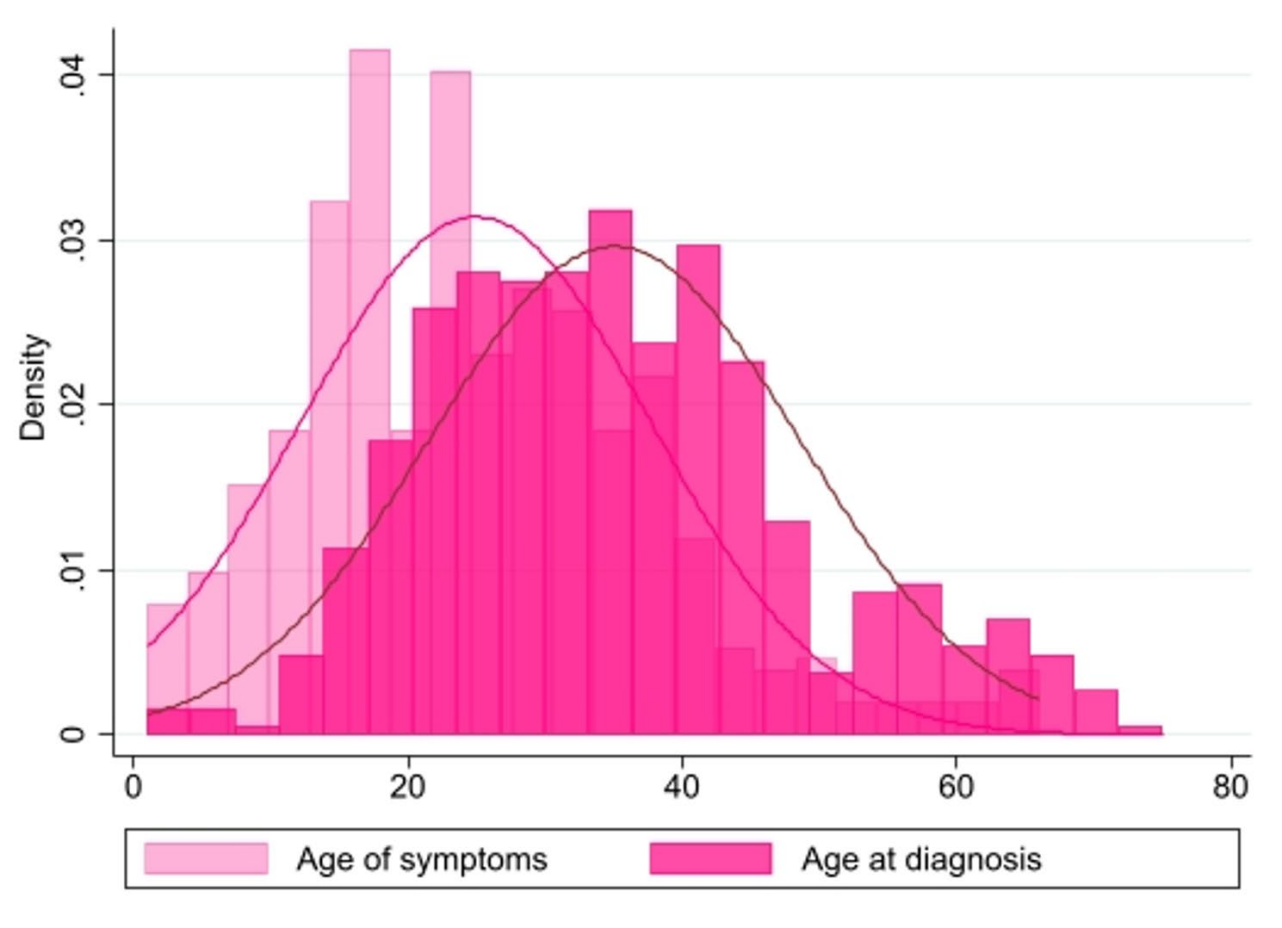
Age distribution of symptom onset and diagnosis demonstrating diagnostic delay. The histogram displays the density distributions and the overlaid normal distribution curves of age at first symptom onset (light pink) and age at diagnosis (dark pink) across the study population (*n* = 575). The x-axis represents age in years (0–80), and the y-axis represents density of observations

The LAM-PREP dataset revealed striking diagnostic delays that significantly impact patient outcomes. Across all age groups, I observed a mean delay of 10.1 years between self-reported symptom onset (mean age 25.0 ± 12.7 years) and diagnosis (mean age 35.1 ± 13.5 years), representing 28.9% of the mean age at diagnosis (Fig. 1; Table 2). Multiple age groups demonstrated a distinct delay between symptom onset and diagnosis, suggesting that this temporal gap is not merely attributable to historical limitations in diagnostic capabilities or disease recognition among older patient cohorts. The youngest cohort (under 35) experienced the shortest mean diagnostic delay at 4.5 years. The delay increased with age: individuals aged 35–44 faced an average delay of 5.8 years and those aged 45–54 had an 8.6-year delay. Notably, participants aged 55 and older demonstrated the longest delay at 14.3 years, which is more than three times the delay observed in the youngest cohort, with symptoms first appearing at a mean age of 29.5 years and diagnosis not occurring until a mean age of 43.8 years. When expressed as a percentage of age at diagnosis, the delay was 25.4% for those under 35, 23.2% for the 35–44 age group, 25.2% for those 45–54, and reached a maximum of 32.6% for individuals 55 and older.

### Educational attainment affects diagnostic delay

Educational attainment emerged as a strong predictor of diagnostic delay. Among 566 respondents, two-thirds reported having earned at least a bachelor’s degree (377, 66.6%), and one-third held a graduate degree (188, 33.2%) [8]. Given this high level of educational attainment, I hypothesized a correlation between education level and age at diagnosis. Controlling for age and disease severity, multiple regression analysis confirmed that individuals with a bachelor’s degree were diagnosed almost 5 years earlier than those without a bachelor’s (adjusted coefficient − 4.73 years, 95% CI: -7.44 to -2.04, *p* = 0.001). Similarly, individuals with a bachelor’s degree reported symptom onset 3 years earlier compared to the reference group (coefficient − 3.11 years, 95% CI: -5.82 to -0.403, *p* = 0.024).

### Treatment utilization is modified by educational attainment

While mTOR inhibitors can effectively stabilize lung function in LAM, our analysis reveals significant disparities in treatment utilization. Despite being the FDA-approved standard of care, only 53% of patients reported current use of mTOR inhibitors. Educational attainment was a significant predictor of treatment uptake. In a multiple logistic regression model controlling for age and disease severity (Table 3**)**, patients with a bachelor’s degree had 1.75 times higher adjusted odds of using mTOR inhibitors (AOR 1.75, 95% CI: 1.13–2.73, *p* = 0.013), while those with a graduate degree had 2.21 times higher odds (AOR 2.21, 95% CI: 1.41–3.45, *p* = 0.001). In contrast, no such relationship was observed between education level and the use of supplemental oxygen.

## Discussion

My findings reveal several critical gaps in the delivery of care for individuals with LAM. First, patients experience prolonged diagnostic delays averaging nearly a decade, which represent close to one-third of a typical patient’s pre-diagnosis lifespan. This diagnostic lag is especially consequential given that early intervention with mTOR inhibitors can stabilize lung function and slow disease progression [1, 10].

This staggering lag underscores the urgent need to address diagnostic disparities to improve outcomes. Second, these delays appear to disproportionately affect individuals with lower educational attainment, pointing to systemic inequities in disease recognition and healthcare access. While awareness of LAM has improved in recent years leading to more timely diagnoses [9], the data suggest that heightened clinician sensitivity to the socioeconomic context of patients could further reduce diagnostic delay. Disparities in both the timeliness of diagnosis and treatment uptake likely reflect several contributing factors. Patients with lower educational attainment may be less likely to recognize the significance of symptoms [6, 11] or may lack the health literacy required to navigate complex treatment decisions [12, 13]. At the same time, healthcare providers may demonstrate unconscious bias in overlooking disease symptoms in such patients or inadequately communicate the benefits of mTOR inhibitors [14, 15]. Conversely, patients with higher education levels may possess the resources to access treatment more readily, sometimes even when such interventions may not yield meaningful clinical benefit [16]. In contrast to mTOR inhibitor use, no relationship was observed between education level and the use of supplemental oxygen, suggesting that mTOR inhibitors uptake may be mediated by socioeconomic factors beyond clinical need alone. These findings underscore the need for targeted interventions to address healthcare disparities in LAM care.

The strengths of the study include the large sample size and the diversity of the respondents with disease characteristics reflective of the general LAM population. Parsimony was prioritized in model selection to balance explanatory power, interpretability, and statistical stability. Adding income, race, and insurance status variables contributed incrementally to model fit and introduced sparsity issues. Education effects remained stable across specifications, and likelihood-based criteria (AIC/BIC) favored the reduced model including education, age group, and disease severity. By retaining only variables that were both substantively relevant and strongly associated with mTOR inhibitor use, we achieved a model that is robust, interpretable, and less prone to overfitting. It is important to acknowledge certain limitations when interpreting these results. First, a potential for selection bias exists for respondents with internet access, digital literacy, and English fluency. Second, because the study depended on self-reported data, the results, especially regarding subjective variables such as symptom onset, are susceptible to recall bias. Third, the high educational level of the respondents, with a significant majority holding at least a bachelor’s degree, may limit the generalizability of the conclusions to the entire LAM patient population, and may bias the results towards underestimating the disparities in LAM diagnosis and treatment. Finally, unmeasured confounding variables, such as access and availability of specialist care and proximity to LAM clinics, may affect causal interpretation.

## Conclusions

This research highlights a critical and unexplored area of LAM research: the role of socioeconomic factors, particularly level of educational attainment, in shaping LAM patient outcomes, such as timeliness of diagnosis and treatment utilization. Targeted interventions are needed to mitigate provider bias and to enhance patient support across educational and socioeconomic backgrounds, improving diagnostic timing and optimizing treatment utilization for all individuals living with LAM.

**Table 1:** Highest level of education of survey participants

Total number of participants ^a^	575	(100.0)
High school	49	(8.5)
Technical/trade/vocation training	35	(6.1)
Some college	65	(11.3)
Associate degree	40	(7.0)
Bachelor’s degree	189	(32.9)
Master’s degree	149	(25.9)
Doctorate or doctoral-level professional degree	39	(6.8)
Other	9	(1.6)

^*a*^ *Values are given as number and (%) of participants*

**Table 2:** Diagnostic delay of LAM by age group

Age Group	Mean Age at Symptoms Onset (years)	Mean Age at Diagnosis (years)	Diagnostic Delay (years)	Delay as % of Age^a^
under 35	13.2	17.7	4.5	25.4%
35–44	20.2	25.0	5.8	23.3%
45–54	25.5	34.1	8.6	25.2%
55 and older	29.5	43.8	14.3	32.6%
**Overall**	**25.0**	**35.1**	**10.1**	**28.9%**

^a^ *Delay as percentage of mean age at diagnosis*

**Table 3:** Association between education level and mTOR inhibitor use in patients with LAM (adjusted for age and severity^*a*^)

Education Level	Adjusted Odds Ratio (AOR)	95% Confidence Interval	*p*-value	Significance
Less than bachelor’s ^b^	1.00 (Reference)	—	—	—
Bachelor’s degree	1.75	1.13–2.73	0.013	*
Graduate degree^c^	2.21	1.41–3.45	0.001	**

* p < 0.05, ** p < 0.01

^a^ mild - lung function (FEV1) > 70%; moderate - lung function (FEV1 or DLCO) between 50–70%, severe - lung function (FEV1 or DLCO) < 50%

^b^ high school, technical/trade/vocation training, some college or associate degree

^c^ Master’s, doctorate or doctoral-level professional degree

## Data Availability

The data is available upon request.

## Abbreviations

DLCO: Diffusing capacity of the lungs for carbon monoxide
FDA: U.S. Food & Drug Administration
FEV1: Forced expiratory volume
LAM: Lymphangioleiomyomatosis
mTOR: Mechanistic target of rapamycin
TSC: Tuberous Sclerosis Complex
